# Novel insights into mechanisms for Pak1-mediated regulation of cardiac Ca^2+^ homeostasis

**DOI:** 10.3389/fphys.2015.00076

**Published:** 2015-03-17

**Authors:** Yanwen Wang, Hoyee Tsui, Emma L. Bolton, Xin Wang, Christopher L.-H. Huang, R. John Solaro, Yunbo Ke, Ming Lei

**Affiliations:** ^1^Department of Pharmacology, University of OxfordOxford, UK; ^2^Faculty of Life Science, University of ManchesterManchester, UK; ^3^Physiological Laboratory, University of CambridgeCambridge, UK; ^4^Department of Physiology and Biophysics, University of IllinoisChicago, IL, USA

**Keywords:** Pak1, Ca^2+^ homeostasis, heart

## Abstract

A series of recent studies report novel roles for Pak1, a key member of the highly conserved family of serine-threonine protein kinases regulated by Ras-related small G-proteins, Cdc42/Rac1, in cardiac physiology and cardioprotection. Previous studies had identified Pak1 in the regulation of hypertrophic remodeling that could potentially lead to heart failure. This article provides a review of more recent findings on the roles of Pak1 in cardiac Ca^2+^ homeostasis. These findings identified crucial roles for Pak1 in cardiomyocyte Ca^2+^ handling and demonstrated that it functions through unique mechanisms involving regulation of the post-transcriptional activity of key Ca^2+^-handling proteins, including the expression of Ca^2+^-ATPase SERCA2a, along with the speculative possibility of an involvement in the maintenance of transverse (T)-tubular structure. They highlight important regulatory functions of Pak1 in Ca^2+^ homeostasis in cardiac cells, and identify novel potential therapeutic strategies directed at manipulation of Pak1 signaling for the management of cardiac disease, particularly heart failure.

## Introduction

Protein kinases are versatile signaling molecules involved in the regulation of a wide range of physiological processes. Of these, the p21-activated kinases (Paks) form a group of serine/threonine protein kinases activated by Cdc42 and Rac1, and were first discovered in rat brain tissue two decades ago (Manser et al., 1994). The structure, substrate-specificity and functional role of Paks are evolutionarily conserved from protozoa to mammals. Vertebrate Paks are particularly important in cytoskeletal remodeling and the assembly of focal adhesion structures, thereby contributing to the modulation of dynamic processes such as cell migration and synaptic plasticity (Manser and Lim, 1999; Hofmann et al., 2004; Zhao and Manser, 2005). Over the past decade, significant progress has been made in understanding the functions of Pak1, a key member of the Pak family, in particular, its roles in the regulation of excitability and contractility of the heart (Ke et al., 2004, 2007, 2009). The present review now provides an updated account of these recent findings regarding additional roles of Pak1 in Ca^2+^ homeostasis and Ca^2+^ handling in cardiac cells.

## Regulation of L-type Ca^2+^ channels in sinoatrial node pacemaker cells

The role of pak1 in the regulation of ion channel activity in cardiomyocytes was first demonstrated in isolated guinea pig sinoatrial node (SAN) pacemaker cells (Ke et al., 2007). In cultured guinea pig SAN cells, where active Pak1 expression was induced through infection with recombinant adenovirus expressing constitutively active Pak1 (CA-Pak1), responses of both L-type Ca^2+^ channel (LTCC) and delayed rectifier K^+^ channel currents to β-adrenergic stimulation by isoproterenol were depressed in comparison to SAN cells infected with control virus, Ad-LacZ. Similarly chronotropic responses to isoproterenol stimulation, reflected in repetitive action potential frequency were depressed in both intact SAN tissue and isolated SAN cells expressing active Pak1, when compared to controls expressing Ad-LacZ (Ke et al., 2007). In contrast, Pak1 deletion in cardiac specific conditional knockout (Pak1cko) or in total knockout mice resulted in an increased SAN driven heart rate (Wang et al., 2014a). These modified responses to isoproterenol stimulation in SAN tissue and cells infected by CA-Pak1 likely reflect alterations in protein phosphorylation which modulate LTCC and delayed rectifier K^+^ channel activity, and their responses to β-adrenergic stimulation.

Our further studies focussing on LTCC regulation in SAN cells implicated Pak1 as a regulator of protein phosphatase 2A (PP2A). Immunoprecipitation studies indicated that Pak1 and PP2A form a complex, leading to the hypothesis that Pak1 may regulate LTCC activity through PP2A action (Ke et al., 2007). This hypothesis was substantiated by studying the influence of the PP2A inhibitor okadaic acid (OA) on the effects of isoproterenol on LTCC activity in Ad-Pak1–infected cells. OA partially reversed the suppressant effect of active Pak1 on the response of LTCCs to isoproterenol in Ad-Pak1–infected cells. This suggests that Pak1 acts by increasing PP2A activity. Conversely, our recent study demonstrated that mice with a cardiomyocyte-specific Pak1 deletion (Pak1cko) showed higher heart rates than their control littermates (Pak1f/f), although Pak1cko and control Pak1f/f mice showed similar baseline electrocardiographic P wave durations and P-R, QRS and QT intervals (Wang et al., 2014a).

Accumulating evidence implicates a coordinated interplay between the activities of kinases and phosphatases in modulation of LTCC-mediated Ca^2+^ influx even in the absence of humoral stimulation. For example, in the well-known β-adrenergic receptor/protein kinase A (PKA) cascade, inhibitor-1 is a downstream PKA target whose activation results in an attenuation of protein phosphatase 1 (PP1) activity (Gupta et al., 1996). Santana et al. (2002) showed that the phosphatase calcineurin opposes PKA action in mouse ventricular myocytes. Application of the PP2A inhibitor OA can activate LTCC (duBell and Rogers, 2004). Calyculin A, which inhibits both PP1 and PP2A, increases contractility in ventricular myocytes by increasing LTCC activity (duBell et al., 2002). A complementary study by duBell et al reported that addition of exogenous PP2A decreased LTCC currents in rat ventricular myocytes (duBell et al., 1996).

These results together suggested an existence of a dynamic regulatory balance between kinase and phosphatase activity in regulating the LTCC and delayed rectifier K^+^ channel activity in cardiac cells that may be important in controlling cardiac pacemaker activity in response to autonomic and humoral stimulation.

## Regulation of Ca^2+^ handling and Ca^2+^ homeostasis in ventricular myocytes

In parallel with Pak1 action in SAN myocytes, enhanced Pak1 function brought about by Pak1 activating peptide, PAP (Wang et al., 2014b) in ventricular tissue prevented hypertrophic associated ventricular arrhythmias, and Pak1 deletion in *Pak1*^cko^ or in knockout mice increased the risks of ventricular alternans and arrhythmias compared to WT mice. These findings went with a co-immunoprecipitation of Pak1 and PP2A suggesting a complex formation in ventricular myocytes, in common with SAN cells (Ke et al., 2007). Recent studies have also suggested regulatory roles of Pak1 in Ca^2+^ homeostasis in ventricular myocytes (Sheehan et al., 2009; DeSantiago et al., 2013; Wang et al., 2014a). CA-Pak1 over-expression altered Ca^2+^ transient decay constants (τ_Ca_) (Sheehan et al., 2009) and antagonized adrenergic signaling by attenuating isoproterenol-induced increases in the activity ofLTCCs and other proteins regulating Ca^2+^ handling (Sheehan et al., 2009). In contrast, ventricular myocytes from *Pak1*^cko^ mice with a cardiomyocyte-specific *Pak1* knockout showed abnormal Ca^2+^ homeostasis including increased diastolic [Ca^2+^]_i_, as well as decreased sarcoplasmic reticular (SR) Ca^2+^ content and decreased SERCA function, particularly during β-adrenergic stress (Wang et al., 2014a). Significant differences in Ca^2+^ homeostasis were observed between isolated *Pak1*^cko^ and wild type, *Pak1*^f/f^, ventricular myocytes. Diastolic [Ca^2+^]_i_ was higher in *Pak1*^cko^ than *Pak1*^f/f^ myocytes under both baseline and chronic β-adrenergic stress conditions. *Pak1*^cko^ myocytes showed more frequent irregular, alternating Ca^2+^ transients and/or Ca^2+^ waves at increased stimulation frequencies than *Pak1*^f/f^ myocytes under both baseline and chronic β-adrenergic stress conditions during 0.5, 1, and 3 Hz field stimulation. This abnormal Ca^2+^ homeostasis in *Pak1*^cko^ myocytes correlated with differences in evoked cytosolic and SR Ca^2+^ responses between *Pak1*^f/f^ and *Pak1*^cko^ myocytes, in both the absence and presence of chronic β-adrenergic stress (Wang et al., 2014a). Thus, time constants for decays of the Na^+^-Ca^2+^ exchange (NCX) current (*I*_NCX_) following *I*_NCX_ induction by 10 mM caffeine were significantly greater in *Pak1^cko^* than *Pak1*^f/f^ myocytes under chronic β-adrenergic stress. SR Ca^2+^content, measured by integration of the *I*_NCX_ records, was reduced in *Pak1*^cko^ compared to *Pak1*^f/f^ myocytes in both the absence and presence of chronic β-adrenergic stress. The decay rate constants of systolic Ca^2+^ transients in *Pak1*^cko^ myocytes *k*_SERCA_, representing the kinetics of cytosolic Ca^2+^ removal brought about by both SERCA and NCX, was significantly lower than that shown by *Pak1*^f/f^ myocytes in both the absence and presence of chronic β-adrenergic stress (Wang et al., 2014a). The rate constant *k*_SERCA_ was reduced by chronic β-adrenergic stress in both the *Pak1*^f/f^ and *Pak1*^cko^ myocytes but more severely so in the *Pak1*^cko^ myocytes. Finally, peak systolic [Ca^2+^]_i_, estimated from the differences between peaks and baselines of systolic Ca^2+^ transients obtained during regular stimulation, was indistinguishable between *Pak1*^f/f^ and *Pak1*^cko^ under baseline conditions, but was significantly reduced by chronic β-adrenergic stress in both *Pak1*^f/f^ and *Pak1*^cko^, and again more severely so in the *Pak1*^cko^ (Wang et al., 2014a). In contrast, LTCC activity was indistinguishable between *Pak1*^cko^ and *Pak1*^f/f^ under both baseline and chronic β-adrenergic stress conditions, but chronic β-adrenergic stress reduced LTCC current in both *Pak1*^cko^ and *Pak1*^f/f^ ventricular myocytes, which suggests that genetic deletion of Pak1 did not alter the expression and activity of LTCC in these cells. This result contrasts with the effect of CA-Pak1 in SAN cells, and may reflect differing effects of Pak1 on ion channels in different cell types or physiological conditions, and requires further investigation (Wang et al., 2014a). These alterations in Ca^2+^ homeostasis were associated with an increased incidence of ventricular arrhythmias and electrophysiological instability during either acute or chronic β-adrenergic challenge induced by isoproterenol in *Pak1^cko^* hearts. Hence modulation of Pak1 activity modified Ca^2+^ handling under both physiological and β-adrenergic challenge conditions.

Our further molecular studies associated these physiological findings with an impaired SERCA2a function and down-regulation of SERCA2a mRNA and protein expression in *Pak1*^cko^ hearts (Wang et al., 2014a). Further exploration of this altered transcriptional regulation in cultured neonatal rat cardiomyocytes (NRCMs) demonstrated that exposure to control Ad-shC2 virus infection increased SERCA2a protein and mRNA levels following phenylephrine challenge (Wang et al., 2014a). This was abolished by the Pak1-knockdown in Ad-shPak1-infected NRCMs and increased by constitutive over-expression of active Pak1 (Ad-CAPak1) (Wang et al., 2014a). This regulation appeared to involve activation of serum response factor (SRF), a transcriptional factor well-known for its vital role in regulation of cardiogenesis genes in the Pak1-dependent regulation of SERCA2a (Wang et al., 2014a).

The above results indicate that modulation of Pak1 activity in ventricular myocytes can have a significant impact on Ca^2+^ handling in these cells under both baseline physiological and β-adrenergic challenge conditions.

## Possible roles in maintaining transverse (T)-tubular structure

Preliminary evidence (DeSantiago et al., 2013) suggests structural roles of Pak1, in addition to the above functional role of Pak1 in maintaining transverse (T)-tubular structure, which is altered in hypertrophic remodeling. Pak1^−/−^ ventricular myocytes showed decreased cell capacitances compared to WT suggesting decreased T-tubular density. Under these conditions, cells showed comparable SR Ca loads and phospholamban phosphorylation, while their systolic Ca^2+^ transients showed decreased amplitudes and delayed rise times, consistent with a reduced coupling between LTCC-mediated Ca^2+^ influx and RyR2-mediated Ca^2+^-induced Ca^2+^ release (CICR); changes which were not observed in Pak1^−/−^ atrial myocytes. Such findings are consistent with the central role of T-tubules in triggering and synchronizing excitation–contraction coupling, and merit further exploration. T-tubular remodeling has been reported in other cardiac pathologies, and could well be involved in the associated alterations in cellular processes, and hence cardiac function.

**Figure 1:**
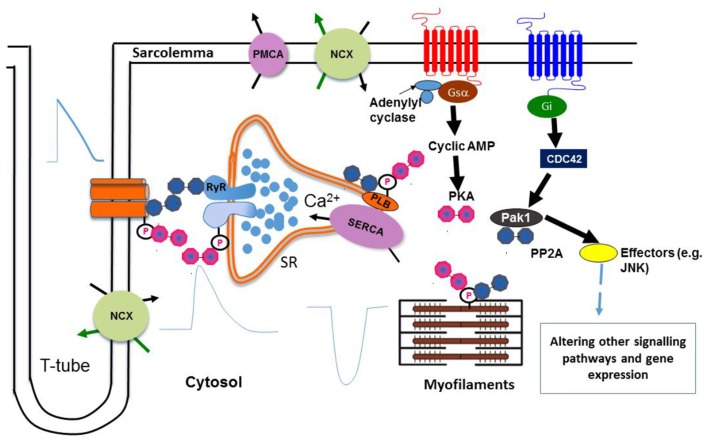
**Regulation of Ca^2+^ homeostasis by Pak1**. Protein kinase PKA and phosphatase PP2A are associated with key Ca^2+^ handling and regulatory proteins, which in turn are linked to upstream signaling cascades. A balance of protein kinase and phosphatase activities is required to maintain normal cardiac functions. Pak1 also regulates hypertrophic signaling and gene expression of SERCA2a through other signaling pathways by activating its downstream effectors (e.g., JNK). (Liu et al., 2011; Wang et al., 2014a) (NCX: Na^+^-Ca^2+^ exchanger, PMCA: Plasma membrane Ca^2+^ ATPase, JNK: c-Jun N-terminal Kinase).

In summary, as illustrated in Figure 1, these novel Pak1 effects on Ca^2+^ homestasis complement previously established actions upon PP2A and the resulting balance between kinase and phosphatase activity in controlling cardiac ion channel activity and rhythmic Ca^2+^ cycling.

Earlier reports (Liu et al., 2011) had identified Pak1 as a novel regulator of hypertrophic remodeling whose cardiomyocyte-specific deletion exacerbated cardiac hypertrophy leading to heart failure, following transverse aortic constriction. These features were prevented by the non-selective Pak1 activator FTY720 in wild-type but not Pak1^cko^ mice (Liu et al., 2013). Such cardiac hypertrophy, with improved cardiac function and decreased myocyte apoptosis compared to WT, was reduced in mice over-expressing Pak1, with improved cardiac function and decreased myocyte apoptosis compared to WT (Mao et al., 2009). A recent study identified Pak1 as one of the significant genes on the core networks for dilated cardiomyopathies by pathway analyses in a Genome wide association study (GWAS) dataset of patients suffering from DCM (Backes et al., 2014). Over the past decade, significant progress has also been made in understanding of additional roles of Pak1 in the regulation of Ca^2+^ homeostasis and Ca^2+^ handling in turn regulating cardiomyocyte excitability and contractility (Ke et al., 2004, 2007, 2009). There have also been speculative suggestions for a role of Pak1 in the maintenance of transverse (T) tubule structure. The present review now provides an updated account of these recent findings. Thus, Pak1 may thus offer a novel therapeutic target for modulation of Ca^2+^ handling in cardiac disease conditions.

### Conflict of interest statement

The authors declare that the research was conducted in the absence of any commercial or financial relationships that could be construed as a potential conflict of interest.
